# Issues navigating the healthcare delivery system among caregivers of children and youth with special healthcare needs: a qualitative study

**DOI:** 10.1186/s12913-026-14931-2

**Published:** 2026-06-11

**Authors:** Caitlin M. Meyer, Rebecca K. Campbell, Maura R. Kelleher, Ebonie Zielinski, Molly Hofmann, Jessica D. Rothstein

**Affiliations:** 1 Division of Community Health Sciences, School of Public Health, University of Illinois Chicago, 1603 W. Taylor Street, Chicago, IL 60612 USA; 2 Division of Epidemiology & Biostatistics, School of Public Health, University of Illinois Chicago, 1603 W. Taylor Street, Chicago, IL 60612 USA; 3 Division of Specialized Care for Children, University of Illinois Chicago, 3135 Old Jacksonville Road, Springfield, IL 62704 USA; 4 Division of Specialized Care for Children, University of Illinois Chicago, 4700 N. Sterling Avenue, Peoria, IL 61615 USA

**Keywords:** CYSHCN, Caregiver burden, Care coordination, Health services

## Abstract

**Introduction:**

Caregivers of children and youth with special health care needs (CYSHCN), including those with disabilities, have numerous roles managing care for their children. While caregiver burden is well documented, less is known about how barriers within the healthcare delivery system contribute to this burden and how systems can be redesigned to better support families. This study explored Illinois caregivers’ experiences navigating the healthcare delivery system for CYSHCN and sought their perspectives on resources needed to reduce caregiver burden.

**Method:**

We conducted a qualitative study as part of a statewide needs assessment in Illinois. Three focus group discussions were conducted with caregivers of CYSHCN recruited through partner organizations. Discussions included participatory activities to elicit priority health needs and concerns for families of CYSHCN in Illinois. Audio recordings were transcribed verbatim, coded, and analyzed by the research team using content analysis to identify themes related to systems-level drivers of caregiver burden and potential solutions.

**Results:**

Caregivers (*n* = 22) described substantial emotional, logistical, and financial burden arising from fragmented healthcare delivery and insurance systems. Key challenges included poor communication and coordination across providers and health systems, inconsistent electronic health record integration, limited access to pediatric specialists, and inadequate availability and reliability of in-home nursing care. Insurance-related barriers, including instability in coverage, prior authorization requirements, and gaps in coverage for needed supplies, further exacerbated caregivers’ strain. Despite these challenges, participants also identified approaches, resources, and supports they have utilized that mitigate caregiver burden, such as multidisciplinary care models, family-centered providers, and support networks. Caregivers emphasized the need for expanded care coordination and navigation, increased mental health supports for families, more flexible insurance and reimbursement policies, and greater investments in home-based care.

**Discussion:**

The findings from this study underscore the need for improvements to the healthcare delivery system through coordinated efforts. Health systems and payers should prioritize the expansion of family-centered care coordination, strengthen communication across providers, and invest in a more stable home healthcare workforce. Advancing disability-inclusive, family centered systems through these strategies is essential to fostering better outcomes for both caregivers and CYSHCN.

**Supplementary Information:**

The online version contains supplementary material available at 10.1186/s12913-026-14931-2.

## Background

Adequate support of children and youth with special health care needs (CYSHCN) relies not only on clinical care, but also on informal caregivers who attend to them every day. CYSHCN are defined as children up to 17 years old who have or are more likely to develop a chronic developmental, physical, emotional, or behavioral condition and require more healthcare services than children in general [1]. This population encompasses a wide range of conditions, including more common health issues such as developmental delay and hearing problems, as well as highly complex medical conditions such as cerebral palsy and congenital heart defects [2]. Among CYSHCN, those with highly complex needs may also experience significant physical, cognitive, or behavioral limitations that rise to the level of disability and affect the child’s ability to participate in daily activities. The complexity of CYSHCN with disabilities can extend beyond their management of a medical condition to include substantial family-identified service needs such as coordination of specialty care, access to assistive technology, educational supports, or community-based supports [3]. Family caregivers play a critical and multifaceted role in supporting CYSHCN, not only managing daily home care such as administering medications, feeding, and bathing, but also serving as their children’s de facto care coordinators and medical advocates [4].

For many caregivers, these experiences are deeply rewarding, leading to a heightened sense of purpose, personal growth, and stronger family bonds [5]. Nevertheless, caregivers often face significant challenges due to the demanding nature of their responsibilities such as high levels of demands on their time from scheduling and attending appointments with multiple providers [6], financial strain due to services not covered by insurance, loss of formal work [7], and social isolation [8]. These challenges, collectively described as “caregiver burden,” have been associated with negative mental and physical health outcomes for the caregivers [7–11]. For example, Cohn and colleagues [9] found that parents of chronically ill children had a 75% increased risk of depression and 40% increased risk of anxiety compared to parents without chronically ill children. Additionally, scholars have found that caregivers of CYSHCN experience stress [7], anger, feelings of helplessness, and low energy [8], chronic pain [10], inflammatory dysregulation [11], and cardiovascular disease [9]. Poor emotional and physical health among caregivers can also negatively affect their children’s health and development [10].

**Table 1:** Participant characteristics, *n* = 22

Characteristics	*n* (%)
**Age (years)**	
30–39	6 (27.3)
40–49	10 (45.4)
50–59	6 (27.3)
60+	0 (0)
**Gender Identity**	
Woman	17 (77.3)
Man	5 (22.7)
Non-binary, gender queer, transgender, or gender non-conforming	0 (0)
**Race/Ethnicity**	
Hispanic, Latino, Latina	3 (13.6)
White	15 (68.2)
Black or African American	2 (9.1)
Asian, Asian American, or Pacific Islander	2 (9.1)
American Indian, Native American, Indigenous	0 (0)
**Marital Status**	
Married or domestic partner	21 (95.5)
Single, never married	1 (4.5)
Separated, divorced, widowed	0 (0)
**Highest Level of Education**	
Less than a high school degree/GED	0 (0)
High school degree/GED and some college	3 (13.6)
Associate degree	2 (9.1)
Bachelor’s degree	6 (27.3)
Graduate degree	11 (50)
**Geographic Region**	
Cook County	11 (50)
Large suburban counties	6 (27.3)
Medium/small urban counties	1 (4.5)
Rural counties	4 (18.2)
**Employment Status**	
Employed full-time	10 (45.5)
Employed part-time	3 (13.6)
Homemaker	6 (27.3)
Not employed	1 (4.5)
Self-employed	2 (9.1)
Other	0 (0)
**Household Member with Medicaid**	
Yes	11 (50)
No	10 (45.5)
Prefer not to answer	1 (4.5)
**Ages of CYSHCN** (*n* = 27)	
1 to 5 years	2 (7.4)
6 to 12 years	6 (22.2)
13 to 19 years	16 (59.3)
Older than 19 years	3 (11.1)
**Total Number of Children Responsible For** (*n* = 21)	
1	3 (14.3)
2	8 (38.1)
3	8 (38.1)
4+	2 (9.5)

The consequences of caregiver burden are widespread, as an estimated 14.1 million adults in the U.S. cared for a child with special needs under the age of 18 in 2019 [1, 12]. In 2019–2020, an estimated 19.4% of U.S. children had a special health care need [1], including 18.9% of Illinois children [13]. With recent advancements in medical care and technology and increased life expectancies, the number of CYSHCN who require dedicated support from primary caregivers will continue to increase [14, 15].

Previous studies have identified several factors associated with negative health consequences for caregivers of CYSHCN. Limited research, however, has explored caregivers’ personal experiences navigating complex healthcare and social service systems. There is a need to better understand how these systems contribute to caregiver burden and to identify opportunities to alleviate logistical, emotional, and financial strains. This qualitative study explored Illinois caregivers’ experiences interfacing with the healthcare delivery system for CYSHCN and sought their perspectives on the types of resources needed to reduce caregiver burden.

## Methods

### Study design

This qualitative study was carried out cross-sectionally as part of a larger statewide needs assessment of health concerns in Illinois. Focus group discussions (FGDs) were conducted as part of a broader effort to understand the priority health concerns for CYSHCN in Illinois. This study was implemented as part of an ongoing academic-practice partnership between a team of maternal and child health researchers and the Illinois Department of Public Health. The partnership, which was formalized more than a decade ago, primarily entails the academic partner providing analytic capacity and expertise to support state-led priorities and projects.

### Study setting

This study was conducted with families across the state of Illinois, which includes a mix of urban, suburban, and rural areas. The majority of Illinois counties are classified as nonmetropolitan based on federal urban-rural classification systems [16], yet most of the population resides in metropolitan areas. Care for CYSHCN with disabilities often involves multiple providers and healthcare settings, including subspecialty care, rehabilitation programs, and hospital-based care, which are concentrated in urban centers such as Chicago. Healthcare services for CYSHCN in Illinois are delivered through a combination of public (i.e. Medicaid) and private insurance systems. Some families receive services through Illinois Medicaid Home and Community-Based Service waivers, including the Medically Fragile, Technology Dependent (MFTD) Waiver, which supports individuals who require skilled nursing care to live in a community rather than institutional setting.

### Participants

Study participants were parents of CYSHCN residing in Illinois. Recruitment was carried out in collaboration with partner organizations that serve families of CYSHCN and have active advisory boards or family councils in which community representatives convene. Separate focus groups (*n* = 3) were conducted with members of each board or council to take advantage of established rapport. The research team provided the organizational contacts with a tailored recruitment flyer to disseminate to their boards/councils, giving interested individuals an opportunity to voluntarily participate. Although the partner organizations were not aligned with any single disease, one primarily served parents of children with medical complexity who were recipients of the MFTD Waiver.

### Data collection

FGDs were conducted either in person or virtually via Zoom and were facilitated by two maternal and child health researchers with substantial experience conducting qualitative studies with community members. Discussions were loosely structured by a discussion guide (see Supplementary File) and began with a set of introductory questions, followed by a participatory activity focused on identifying health needs and priorities for CYSHCN. Participants were asked to write down their top 3–5 health concerns, and these ideas were then displayed and grouped thematically using a whiteboard—either a physical board during in-person sessions or Zoom’s virtual whiteboard for remote discussions. Participants contributed through physical notecards or digital “sticky notes,” depending on the format, as well as verbal sharing.

The participatory grouping activity served as a foundation for deeper discussion of the emerging topical areas. This approach helped ensure that all participants’ perspectives were captured. Displaying participants’ contributions on a whiteboard reinforced the value of each individual’s input and promoted an inclusive environment for discussion.

Before concluding each session, participants were asked to complete a brief and anonymous socio-demographic survey. The FGDs lasted an average of 73 min and took place between January and May 2024. Participants received an electronic gift card in recognition of their time and insights.

### Data analysis

Focus groups were audio-recorded and transcribed verbatim by automatic speech recognition software. Transcripts were then reviewed by a member of the research team to correct any transcription errors and to ensure participant anonymity. Three members of the research team with rigorous training in qualitative data analysis applied codes to the transcripts using Dedoose software (SocioCultural Research Consultants, LLC, Los Angeles, CA). The analytic team was selected based on both qualitative research expertise and familiarity with the subject matter. Team members brought a range of perspectives to the analysis, including lived experience as the parent of a young child with special healthcare needs and professional and advocacy experience with CYSHCN and their caregivers.

A codebook consisting primarily of emergent codes from the data, including the grouped responses from the participatory activities, was developed iteratively during the initial phase of coding. The team met regularly to review and refine the codes, definitions, and clarifying examples. After finalizing the codebook, previously coded transcripts were reviewed by two team members to ensure consistency and application of the most current coding framework. Following procedures for content analysis outlined by Hsieh & Shannon, codes were organized into categories, sub-themes, and overarching themes through iterative discussions among the team [17]. Researchers also engaged in ongoing reflexive discussions and memo writing to examine potential biases and support careful interpretation of the data. In addition, the team continuously consulted with state agency-based partners whose work focuses on advocacy and support for CYSHCN to incorporate their perspectives and expertise into the analysis. Illustrative quotes from the FGDs were identified and are included below to provide contextual depth and strengthen the credibility of our findings.

### Ethical approval

The Institutional Review Board at the University of Illinois Chicago designated this study as exempt in accordance with federal guidelines (Reference Number: 2023 − 1455). All study procedures were conducted in accordance with institutional guidelines and the Declaration of Helsinki. Verbal informed consent for participation and for the audio-recording were obtained from all participants prior to the start of each FGD. Clinical trial number: not applicable.

## Results

### Participant characteristics

Twenty-two caregivers participated in three FGDs. Participants were between 30 and 59 years old, with a mean age of 44.5 years (Table 1). Most participants were women, non-Hispanic White, held a graduate degree, and were employed full-time. Participants cared for CYSHCN of all ages and with a range of severe conditions, such as spina bifida, hydrocephaly, and kidney failure; more than one-third (36.4%) of participants had children who qualified for the MFTD waiver. Four caregivers cared for multiple CYSHCN, and approximately half of participants had a household member with Medicaid.

### Healthcare system issues leading to caregiver burden

Participants frequently shared frustrations related to communication and coordination within the healthcare system. The need to navigate care among multiple specialists within different hospital systems and cities proved time-consuming and exhausting for parents. One mother spoke of the increased burden over the years:

My daughter has specialists both locally and out of state in a variety of different places, so it’s like three main hospitals… It is a lot of coordination I would say. When she was younger, we had her [primary care provider] help us a lot with that… now that she’s older, we don’t have that anymore and we’re just using a lot more different systems now, so it ends up being a lot more coordination on our part in the day-to-day.

Seeking care from multiple specialists often leads to communication breakdowns. These challenges place the obligation of communication and coordination on caregivers, leaving them responsible for sharing new information with their child’s clinicians and often repeating the same information in back-to-back appointments. One parent shared,

We are seen through Complex Care… they try and get us two or three [appointments] a day since it’s about an hour from where we live. Not horrible, but still an inconvenience… If we do have three or four appointments in that day at the same hospital, every appointment asks us about the medication, every single appointment.

Relatedly, participants discussed the lack of consistency in electronic health records (EHR) use and the time required for them to share information at every appointment. Several participants discussed that even when the same EHR is used, providers often document patient information in different ways, such that providing the information themselves is easier. A mother shared:

The different hospital systems, even though there’s an underlying database, it’s not copied across all the different platforms, so you have to, every single time you see a new specialist, update all of the medications. Again, those little annoying things, but that adds up when you do it twice a month.

Participants also described how the limited number of pediatric specialists in Illinois complicated care. The demands of frequent visits and extended wait times for appointments and travel time to get to healthcare appointments and medical device providers proves incredibly taxing, especially among families living in rural and suburban areas: *“We have probably 12 different specialists out of four different hospitals and so we’re traveling anywhere from 30–90 minutes to get to an appointment.”* One participant mentioned that the transportation challenges could be eased by having telehealth appointments, but their specialists do not offer this option.

An additional healthcare system issue affecting participants involves the limited reliability and availability of at-home nursing care for children with more complex conditions. Participants shared additional caregiver burden was created from repeatedly switching nursing agencies to find consistent and reliable care and services for their child: *“We as families are just agency jumping. We’re trying to find the best situation*,* who can staff us*,* who can’t. It just adds a lot more work to our plate and constantly advocating and trying to move things forward.”* Caregivers themselves are often responsible for filling the gaps left by inadequate nursing support. Participants shared how in some cases their in-home nurse was a Certified Nursing Assistant (CNA) who had limitations on their scope of practice hindering their ability to do certain tasks, such as changing equipment or administering medications. Parents recalled that they had to leave the workforce as a result, which adds financial pressure for their family, leading to increased stress and anxiety. One parent shared the journey of finding help for her two-year-old son:

I even had some cases where I felt like my son’s safety was in jeopardy in the home because of the care he was receiving… We now have an absolutely fantastic CNA who we love [but] she’s limited in what she can do. I have to be home, I have to help with his feedings, I have to help with medication. So even though we’re getting some help, it’s not quite the level of help that we need.

### Insurance system issues leading to caregiver burden

Participants encountered inconsistent and disjointed coverage from private insurance plans and Medicaid insurance. One of the most common challenges involves sudden and unexpected changes to medication coverage and prior authorization requirements. Multiple caregivers described challenges when they experienced a change in the insurance coverage for a medication their child had been taking for years and that was previously covered by insurance. Oftentimes they ultimately had to pay out-of-pocket for necessary medications following abrupt changes in medication coverage:

I think one of the most frustrating things about that is the lack of knowledge that families have about that as these changes are going in place… If we had known about it ahead of time, there would’ve been a chance for us to navigate that better. But I can’t tell you the number of times that—I’m sure I’m not alone—that we’ve forked over money just because they can’t go without. But how do you do that? It’s not sustainable.

In some cases, insurance does not cover necessary supplies at all, such as diapers for older children and catheters. The need to constantly be in touch with both insurance companies and healthcare providers to advocate for their children and secure essential supplies demands substantial effort from caregivers, compounding their stress:

It’s so frustrating because that kind of maze if you will, occurs with the really important stuff, the big-ticket items, but it also occurs with just the little tiny things like number of syringes you can get each month… Your insurance will say, ‘well no, there’s no limit as long as you have a letter of medical necessity,’ and your doctor is saying, ‘I wrote the script for it.’ There’s nobody other than you to kind of be that person on the phone with all these different people… to get what you need in a timely fashion.

The time required for navigating payment and billing for healthcare and medical devices proved equally draining, as one father mentioned, *“for the billing to make sense… I don’t have a degree in medical billing but apparently you need one.”* Other parents discussed the time spent searching for creative ways to afford special equipment like baths and bicycles that insurance or Home and Community-Based Services (HCBS) waivers would not cover.

Participants also faced issues qualifying for needed HCBS waivers or additional coverage. This was particularly challenging in the case of what some participants described as *“gray children”* who had significant health needs but did not meet the medical requirements for the MFTD waiver or other HCBS waivers. Some families with private insurance described a need for additional coverage due to limitations of their plan but they could not qualify for Medicaid or other HCBS waivers due to an income cap or other eligibility restrictions. At the same time, participants who did qualify for waiver programs faced challenges in receiving additional support from organizations that provide medical equipment or devices due to the organizations assuming that the waiver program provided all of the needed devices. As one parent shared, *“We’ve run into that a couple of times where we want to be honest when we’re filling out the application*,* but when you’re honest it looks like you should be getting everything that you need met and clearly we’re not*,* so that can be a bind.”*

### Approaches, resources, and supports that decrease caregiver burden

Alongside the discussion of the daily challenges experienced due to systemic constraints, participants also described several types of resources and supports both within and outside of the healthcare system that relieve some of their burden. A couple of participants described the holistic approach taken by their child’s care team, which allows them to see multiple specialists on the same day. One mother shared her experience with an out-of-state children’s hospital that has a spina bifida clinic, *“when they do… clinic day*,* they [the specialists] kind of sit in a room together*,* so to speak. They come in and see you and then they all have their reports together where they talk to each other*,* which is nice.”* Others expressed appreciation for providers who consider not only the needs of the child, but also the well-being of the parents. Examples include a child’s provider prescribing a medication for the mother so she would not need to attend an extra appointment, and another considering the mother’s own sleep needs:

And she [the doctor] is like, I’m very concerned that you have to wake up and give him this medication at 2:00 AM every night. Should we just move it to a different time? And so, I appreciate the physicians that are like, ‘I want you to sleep more than he gets this treatment. It can only work if we work as a system.’ And so, I won’t go back to the other ones [doctors], and I will continue seeing those that see us as partners and experts and that actually value the opinions and the observations.

In the community setting, participants spoke about resources available for when devices and equipment were denied by insurance, such as community sites for lending and exchanging equipment. One parent described how a small number of *“lending libraries”* available in the Chicago area offered equipment free of charge, yet she mentioned that *“you have to MacGyver sometimes different supports because it wasn’t designed for your child with a physical therapist or an occupational therapist.”* Other families spoke of obtaining medical beds and medical bicycles from local organizations or families after insurance denied covering these items. Another parent shared online exchange groups such as a *“black market catheter exchange”* for those that cannot afford catheters due to a lack of insurance coverage.

In addition, social support from other caregivers was a key source of comfort and guidance for many participants, with one parent describing other parents of CYSHCN as their *“primary source of go-to knowledge.”* Some families recalled being connected to others through organizations related to their child’s specific illness, both locally and at national conferences. Other caregivers connect through *“parent-formed*,* parent-led”* Facebook groups that are not necessarily disease-specific. One mother shared how the support offered in an online group went far beyond medical questions, encompassing practical and emotional aspects of caregiving:

*We’re all kind of navigating the same systems… so that’s been lifesaving to figure out how to navigate something or where you should go get your haircut that will take a child that can’t sit in a seat*,* things like that. And it’s even just brainstorming with other parents like*,* okay*,* how do we solve this… problem that we’re encountering? And so that’s been helpful.*

### Suggestions to reduce caregiver burden

Participants offered a range of suggestions for alleviating the challenges faced by families with CYSHCN. Several suggestions related to reducing coordination and communication demands on caregivers by providing more support for navigating the healthcare system, such as through a high-quality social worker. One parent provided an example of how an experienced social worker at their son’s hospital was well-equipped to meet their needs: *“Sometimes we don’t even know what we need… she’s [social worker] seen the families going through the NICU experience and having all of these traumatic experiences over and over again that she could anticipate what was coming. So*,* she was able to get ahead of it and provide resources and just the support.”* Others suggested that parents themselves serve as navigators for their peers: *“Parents I think give the best information… Pay us to be the case workers to help other families. I feel like I would give better information.”*

Participants also emphasized the need for mental health resources for the whole family. For many parents, their family’s daily challenges navigating the healthcare delivery system began when their child was born; watching their child go through surgeries and other life-threatening moments and worrying about their child’s future resulted in constant stress and anxiety, sometimes leading to panic attacks. Participants suggested reducing long wait times for mental health care referrals for caregivers and siblings of CYSHCN by issuing referrals directly following a child’s diagnosis:

Even a mental health [referral] with your diagnosis if you’re diagnosed with a chronic condition, just that’s part of the referral strategy standard for the family, not just the individual who’s sick, because we’ve all seen it affects the siblings and the parents. And so if you can get ahead of it…so that you already have a contact point starting sooner rather than when everybody’s drowning.

Additionally, parents expressed a desire for support groups where they could interact with families with similar experiences, suggesting groups coordinated by hospital staff. For example, parents of adolescents participating in a hospital’s youth advisory board, which provided an opportunity for CYSHCN to socialize with others going through similar experiences, recommended similar groups for the whole family.

Finally, participants offered suggestions for policy solutions that could address insurance and loss of income issues. To reduce the inconsistent and disjointed coverage from private insurance plans, one parent expressed, “*For me*,* my big push is Medicaid as a secondary insurance for all kids that have medical complexities*.” Others suggested reimbursement options through Medicaid to pay caregivers to care for their children with medical complexities at home, which would reduce the burden placed on caregivers navigating in-home nursing coverage and improve financial stability.

## Discussion

The current study explored caregivers’ experiences navigating healthcare and social service systems. The findings highlight many challenges that caregivers of CYSHCN face that align with prior literature, including communication and coordination issues [6] and inconsistent and disjointed access to medications and resources [18]. Participants’ narratives shed light on the need for improvements to family-centered care and disability-inclusive coordination and navigation services, healthcare delivery models, caregiving support in the home, and mental and social support resources (Table 2). The results suggest modifications to the healthcare delivery system could alleviate challenges faced by caregivers of CYSHCN.

**Table 2:** Recommended health system strategies to reduce caregiver burden

Key Source of Caregiver Burden	Recommended Health System Strategies
Caregivers must navigate care across multiple providers and systems, often serving as the primary coordinators and intermediaries	Expand family-centered care coordination and navigation programs in accordance with the National Care Coordination Standards for CYSHCN (e.g. shared plans of care, team-based communication)
Frequent appointments across geographically dispersed providers create substantial time and travel burdens for families	Preserve and expand telehealth and hybrid care models for CYSHCN when clinically appropriate; promote care models that consolidate visits (e.g., multidisciplinary clinics)
Inadequate availability and reliability of in-home nursing care shifts extra clinical responsibilities onto caregivers and may lead them to leave the workforce	Strengthen infrastructure for in-home nursing care through workforce development; support caregiver reimbursement and protections for providing home healthcare
Caregivers experience social isolation and rely heavily on informal networks for social support and instrumental support	Integrate support and navigation services within healthcare systems, including caregiver support groups, peer mentoring, and connections to community-based resources; implement a screening tool for caregivers of CYSHCN to identify caregiver burden

The findings illustrate there is an enormous need for expanding and improving family-centered care coordination and navigation within different organizations to relieve caregiver burden among parents of CYSHCN. Disability-inclusive care coordination services may include assistance navigating insurance systems and waivers, facilitating communication across multiple providers, and providing linkages to community resources. The benefits of care coordination have been demonstrated through both observational and intervention studies [19–21]. Turchi and colleagues [21] found that families of CYSHCN using care coordination services had greater odds of receiving family-centered care and decreased odds of having issues with referrals, time coordinating care, and financial burden. A randomized controlled trial found that after six months of placement in a family-centered care coordination program, CYSHCNs’ overall health improved and the number of family needs and personal strain reported by caregivers decreased [19].

The FGDs revealed issues with both the availability and the scope of care coordination services in Illinois. While some study participants shared their positive experiences with care coordination provided through hospital systems or private insurance, many others were not receiving the support they needed. In 2022–2023 in Illinois, 31% of children in need of care coordination did not receive effective services [22]. Care coordination is often offered through medical homes or hospital systems, insurance companies (including Medicaid Managed Care), and community-based programs. In Illinois through Medicaid Managed Care [23], CYSHCN should have care coordination services available to them, however, claims data is used to identify eligible children and this system does not catch all children who may be eligible for or require care coordination services.

Our findings support increased investments to expand care coordination in alignment with the National Care Coordination Standards for CYSHCN put forth by the National Academy for State Health Policy. Two of the framework’s six domains—Shared Plan of Care (SPOC) and Team-Based Communication—offer actionable strategies for addressing key sources of caregiver burden shared by the participants. The SPOC is an individualized care plan that is co-created with families and outlines the child’s medical, developmental, and social goals and maps relevant supports; the SPOC combines evidence-based care from providers with family preferences to produce better care and care coordination for the child [24–26]. The SPOC is shared with everyone on the child’s care team, including their primary care providers, specialists, educators, care coordinators, and other agencies, thereby preventing the need for caregivers to repeatedly share the same information with different providers.

Team-Based Communication complements the SPOC by supporting timely, routine information-sharing among all members of the care team, including families, based on their communication preferences. These team-based models support patients and their caregivers, improve confidence in care received [27, 28], and play a critical role in accessing supports and services [29]. Potential strategies include convening multidisciplinary team meetings, as described by one participant whose child attended a Spina Bifida clinic involving multiple specialists and using secure messaging systems. Improvements in communication systems like these, as supported by the National Care Coordination Standards, would eliminate the reliance on caregivers to serve as intermediaries between providers and reduce the stress of navigating a fragmented system.

Aside from improvements in care coordination and clinician communication, several of the participants suggested and would benefit from expanding the availability of telehealth appointments when physical exams are not necessary. This finding aligns with those from other studies demonstrating that patients appreciate the convenience of telehealth visits [30]. U.S. telehealth regulations expanded during the COVID-19 pandemic [31] and were recently extended through 2027 [32], yet these regulations remain temporary rather than permanent policy. Furthermore, telehealth access is inconsistent due to variation across insurers, state Medicaid policies, and health system-level implementation decisions [30]. The present study underscores the need for policies and health system practices that protect flexible care delivery options, including telehealth, to improve access to care for CYSHCN and reduce caregiver burden [33].

Outside of the traditional healthcare setting, the study findings also point to the need for improvements in the delivery of in-home nursing care. Study participants’ experiences with in-home nursing care reflect what has been termed a “leaky pipeline” in the literature [34]. Issues including insurance coverage gaps, generalized eligibility cut-offs (e.g., for technology dependence), strict hour allocations, and inadequate workforce training often contribute to gaps in home healthcare services, requiring caregivers to provide nursing care to meet their children’s needs [35–37]. Study participants’ reports of having to leave their jobs for these reasons are reflected in findings from other studies indicating that inadequate home health care is associated with significant financial losses for caregivers [38, 39]. There is a clear need for research efforts to examine strategies for improving in-home nursing care and reducing caregiver strain, such as through strengthening workforce education or testing alternative types of caregiving assistance.

Policies oriented towards protections and reimbursements for caregivers providing home healthcare can play an important role in alleviating the challenges. Recently, Illinois has taken important steps in this area through two 2025 amendments: one to the Human Rights Act prohibiting employment discrimination against family caregivers [40] and another to the Medicaid state plan allowing parents who are CNAs to be reimbursed for caring for their medically fragile children at home [41]. Moving forward, researchers should evaluate the extent to which these new provisions not only improve quality of care but also support caregiver well-being. Advocacy efforts are also needed to ensure that future expansions of these amendments consider the needs of caregivers who are not CNAs.

The findings demonstrated that social support and instrumental support are critical for caregivers, similar to findings from Geyer and colleagues [36]. Existing literature has highlighted the benefits of support groups for caregivers of CYSHCN, including online groups [42–44]. Parents in the study had found varying levels of support, often in online support groups; however, many reported finding the sharing of information within the focus groups beneficial and were wanting similar resources. Hospitals and healthcare systems could play a role in facilitating these connections and establishing support groups. One hospital system in the state has a youth advisory board and parents expressed interest in seeing the group expanded to include social support for the full family including siblings, similar to findings from Hickey et al. [45]. Additionally, healthcare systems could utilize peer navigators or parents with more experience to mentor families newer to navigating these intricate systems, suggested in the current study and existing literature [44, 46].

Finally, implementing a screening tool for caregiver burden could help care coordinators or healthcare providers more effectively identify caregivers in need. Several screening instruments have been developed and validated over the past few decades to assess various dimensions of caregiver strain, yet most were originally designed for caregivers of elderly or adult patients [47–49]. One such tool, the Zarit Burden Interview, has been applied to caregivers of children and adolescents with specific chronic conditions [50]. Further research is needed to adapt and validate such instruments for broader use among caregivers of CYSHCN, which would better equip providers or care coordinators to assess caregiver well-being and facilitate timely referrals to support services.

## Limitations

This study may have been limited by the following. First, participants were recruited through collaboration with partner organizations, which may have preferentially reached caregivers already engaging with community resources. However, directly leveraging these organizations may have increased participants’ trust in the study team. Second, the study focused on a single midwestern state, which may limit the generalizability of our findings to caregivers in other geographic and healthcare contexts. Additionally, our sample of participants was relatively homogenous in terms of race/ethnicity and educational achievement. Although we achieved a geographically diverse sample and included caregivers of children with a wide range of special healthcare needs and disabilities, our findings may not reflect the experiences of minoritized populations and other vulnerable groups. Future research should focus on seeking the perspectives of caregivers of color, those from low-income communities, families who do not speak English, and across diverse global contexts.

## Conclusion

Despite these limitations, this study provides valuable insights into how fragmented healthcare and insurance systems place significant logistical, clinical, and financial responsibilities onto caregivers of CYSHCN. Our participants’ experiences also underscored several opportunities for reducing caregiver burden through more family-centered and disability-inclusive systems of care. Such efforts must prioritize care coordination and navigation, improve communication among providers, strengthen the home healthcare workforce, and ensure that insurance and reimbursement systems adequately support families. In addition, healthcare systems should strengthen social support and mental health resources available to all family members of CYSHCN. Advancing family-centered systems through strategies like these is critical to improving both caregiver well-being and outcomes for CYSHCN.

## Supplementary Information

Below is the link to the electronic supplementary material.

Supplementary Material 1

## Data Availability

In order to protect the confidentiality of our participants, our data is not publicly accessible.

## Abbreviations

CNA: Certified nursing assistant
CYSHCN: Children and youth with special healthcare needs
EHR: Electronic health record
FGD: Focus group discussion
HCBS: Home and Community-Based Services
MFTD: Medically Fragile Technology Dependent
SPOC: Shared Plan of Care
