# Supplementary material for: Issues navigating the healthcare delivery system among caregivers of children and youth with special healthcare needs: a qualitative study
Source: BMC Health Serv Res. 2026 Jun 11;26:1149. doi: 10.1186/s12913-026-14931-2 (PMC13488566; doi:10.1186/s12913-026-14931-2)
Supplement: Supplementary file 1 [file 12913_2026_14931_MOESM1_ESM.docx]

Title V Needs Assessment Consumer Focus Groups

**Discussion Guide for Focus Groups (In-Person and Virtual): CYSHCN**

**Introductory Questions**

*Ask the following questions to the group and use probes as needed. Brackets are used to indicate options for population groups to name that may vary depending on the types of participants.*

1. In your own words, how would you describe the community that you live in?
   a) If someone told you that they were moving to your community, what would you tell them about living there?
2. Who are the main healthcare providers for CYSHCN in your community?
3. Aside from healthcare providers, what kinds of organizations and services are there in your community that try to meet the needs of CYSHCN?
   a) What kinds of services or programs do they provide?
   b) Which services or programs have you used in the past or are you currently using?
   c) Are there barriers in your community/lived environment to accessing or taking advantage of any needed services? (this may include broader issues affecting communities such as transportation, housing, or food access)

**Participatory Activity**

*Follow the steps for the participatory activity and integrate discussion questions throughout the activity.*

*For IN-PERSON focus groups: Each participant will be provided with large post-it cards, a sheet of paper, a marker, and a pen.*

*For VIRTUAL focus groups: Participants will be asked to get out a piece of paper and pen, and the facilitator will provide an online interactive whiteboard.*

1. Ask participants to individually write down 3-5 of the most important health issues, concerns, challenges, or needs of CYSHCN that they see in their communities today on a sheet of paper.
2. Ask participants to put a star next to the topic that they believe is the most urgent issue that needs to be addressed.
3. Ask participant to transfer these ideas (details below) so that all participants can view them, and include the star next to the most urgent issue.
   a. *For IN-PERSON focus groups*: Ask participants to write the items on the post-it cards (one item per card). When participants are ready, collect the post-it cards and display them for all participants to see (can use a Sticky Wall or lay them out on a table, as the room allows). Take photos.
   b. *For VIRTUAL focus groups*: Provide a brief demo on how to use the online interactive whiteboard, and then ask participants to write their items on the whiteboard.
4. Participants take a few minutes to look at the different items and which ones have been starred.
5. With input from participants, group the cards together into broader topic areas to the extent possible.
6. Facilitate discussion about why certain items were chosen and the experiences, barriers, resources/services/programs, and priority populations related to these items (see **discussion questions** below).

**Discussion questions for facilitators to integrate during the activity:**

- Are there any **recent experiences** that you’ve had that led you to write down or prioritize this topic/issue? Or experiences of your family members or friends?
- How are the topics/issues that you wrote down **similar to or different from** those shared by other members of the group?
- Are there **resources** available to address this topic/issue? What kinds of services or programs are you aware of?
- If you have used services or programs for this topic/issue, have you been **satisfied** with them? What is **missing or needed**?
- Are there any **groups that face particular challenges** related to this topic/issue? Which groups? Why do you think this is?
- What kinds of **barriers** have you or your family members or friends faced in relation to accessing services or resources for this topic/issue?

**Focused Questions**

*Only if needed, pose these questions to elicit further information about specific topics of interest, based on the topics that have already been discussed during the participatory activity.*

Children/Youth with Special Health Care Needs:

- For parents in need of home care including home nursing for children/youth, has availability and coverage been adequate?
  - What have been the major challenges related to getting home care?
  - Have you faced financial challenges?
- What other support services has your child needed to address other health and social service concerns?
  - Are these services available in your community?
  - Have there been barriers to obtaining these services?
- To what extent are mental health issues a concern for your child?
  - Are the resources available for addressing these issues enough?
  - What else is needed?
  - Are there barriers to accessing available resources/services for mental health issues?
- For those with youth with special health care needs who are transitioning or have transitioned to adult health care services, what have been your experiences with services, support, and care coordination?
  - Have your needs been met?
  - If not, what has been lacking?
  - How could the transition process be improved?
